# Chitosan-Coated SLN: A Potential System for Ocular Delivery of Metronidazole

**DOI:** 10.3390/pharmaceutics15071855

**Published:** 2023-06-30

**Authors:** Simise S. Sikhondze, Pedzisai A. Makoni, Roderick B. Walker, Sandile M. M. Khamanga

**Affiliations:** 1 Division of Pharmaceutics, Faculty of Pharmacy, Rhodes University, Makhanda 6140, South Africa; 2 Division of Pharmacology, Faculty of Pharmacy, Rhodes University, Makhanda 6140, South Africa; p.makoni@ru.ac.za

**Keywords:** solid lipid nanoparticles, SLN, chitosan, mucoadhesion, controlled release, targeted drug delivery, metronidazole

## Abstract

Ophthalmic drops for ocular delivery exhibit inadequate residence time, which often requires multiple daily dosing that may result in patient non-adherence. In this study, the development of a once-daily-dosed chitosan-coated metronidazole (MTZ)-loaded solid lipid nanoparticles (SLNs) for ocular delivery was undertaken. Melt emulsification and ultrasonication were used to manufacture MTZ-loaded SLN, which were subsequently coated with chitosan (CS) by mechanical stirring using a 0.1% *w*/*v* solution. Gelucire^®^ 48/16 and Transcutol^®^ HP were used as the solid lipid and synthetic solvent, respectively, with Tween^®^ 20 included as a stabilizing agent. The critical quality attributes (CQA) of the optimized CS-coated SLN that was monitored included particle size, polydispersity index, Zeta potential, % entrapment efficiency, % MTZ loading, pH, and osmolarity. The optimized coated nanocarriers were evaluated using laser Doppler anemometry (LDA) and were determined to be stable, with particle sizes in the nanometre range. In vitro mucoadhesion, MTZ release and short-term stability, in addition to the determination of the shape of the optimized CS-coated SLN, were undertaken. The mucoadhesive properties of the optimized CS-coated MTZ-loaded SLN demonstrated increased ocular availability, which may allow dose reduction or longer intervals between doses by improving precorneal retention and ocular availability. Overall, our findings suggest that CS-coated MTZ-loaded SLNs have the potential for clinical application, to enhance ocular delivery through the release of MTZ.

## 1. Introduction

Ocular rosacea is a disease that results in inflammation of the surface membranes of the eye, presenting symptoms such as burning or stinging, dryness, and/or blurred vision [1]. Ophthalmic complications such as blepharitis, conjunctivitis, stye formation, perforation keratitis, thinning, corneal neovascularization, marginal corneal infiltration, and ulceration may also occur [2,3]. Ocular rosacea is frequently undiagnosed, as most patients do not present with facial skin aberrations [4], with approximately 90% of patients presenting with neither roseatic skin alteration nor a previous diagnosis [4]. Of the patients who suffer from rosacea, 58% exhibit ocular complications, of which 33% present with corneal involvement [5]. The rate at which ocular complaints occur in patients who have rosacea is approximately 45% to 85% [6,7].

**Figure 1:**
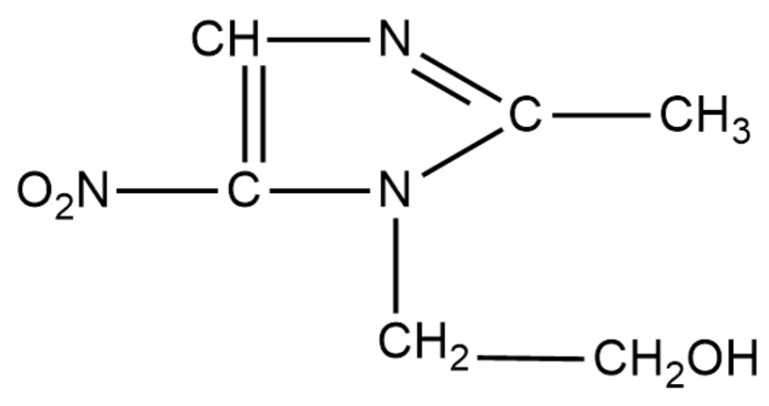
Chemical structure of metronidazole (MW = 171.16 g/mol).

Metronidazole (MTZ) (Figure 1) is a nitroimidazole antimicrobial that exhibits activity against some protozoa and against Gram-negative and Gram-positive anaerobic bacteria [8]. No MTZ containing ocular dosage forms for the treatment of ocular rosacea is currently available [9]. Consequently, in this study, the formulation, development, and manufacture of an ocular delivery system that could sustain the release of MTZ for once-daily application to the eye was undertaken, as this may change the treatment of ocular rosacea. Encapsulating MTZ into solid lipid nanoparticles (SLNs) would permit once-daily application with a formulation designed to minimize adverse effects with the incorporation of generally-regarded-as-safe (GRAS) excipients. Nanoparticles enhance the antibacterial efficacy of the antibiotic, as they penetrate bacterial cells more effectively and deliver the drug to its target more efficiently [10,11]. However, antibacterial studies were not performed. Other SLN formulations have demonstrated retained or improved antibacterial activity [12,13,14].

The use of a 0.75% *w*/*w* metronidazole (MTZ) topical gel resulted in an improvement in rosacea-related eyelid health [15]. The pharmacokinetic properties of MTZ administered in a drop formulation exhibited slow penetration through the lipophilic and hydrophilic epithelial membranes of the cornea, suggesting that this product can only be used for the treatment of infections on the anterior surface(s) of the eye [16], as the topical administration of MTZ reduces neovascularization of the cornea and improves the treatment of ocular rosacea [17].

The use of MTZ as a treatment for ocular rosacea suggests that topical application may be a useful adjunct for treating the condition [15]. MTZ is not only an antimicrobial—it may also exhibit anti-inflammatory and immunosuppressive effects, making it an effective treatment for rosacea, eyelid diseases, and ocular diseases when applied to the margins of the eyelids [15]. Topical MTZ gels may be a safe and effective option for treating adnexal changes that affect the lining and protection of the eye in patients with ocular rosacea [15]. Although the formulation appears to be safe and effective for treating adnexal changes in ocular rosacea, it has not yet been approved by the FDA for ophthalmic use [15].

MTZ is currently available as a 0.75% *w*/*w* gel for application to the skin once or twice a day [9]. Encapsulating MTZ into solid lipid nanoparticles (SLNs) may facilitate the once-daily application of a formulation designed to minimize adverse effects with the incorporation of GRAS excipients. Nanotechnology-based delivery systems have the ability to cross membrane barriers, particularly in the CNS and the gastrointestinal tract [18]. The use of nanotechnology-based delivery systems such as SLN have resolved solubility-related challenges for poorly soluble drugs, while permitting the exploitation of the possibility of overcoming the retinal barrier [18].

Prolongation of the contact time of ocular delivery systems would reduce dosing frequency and facilitate patient adherence. Consequently, chitosan (CS) was used to coat an MTZ–SLN formulation. Chitosan (CS) is a biodegradable, biocompatible, non-toxic compound that is suitable for inclusion in ocular delivery technologies and exhibits mucoadhesive capabilities [19]. The mucoadhesive properties of CS are a function of a positive charge that allows interaction with negatively charged sialic acid in mucin, resulting in the formation of electrostatic bonds that promote adhesion [20]. Chitosan-N-acetylcysteine has been approved for use in Lacrimera^®^, an eyedrop product that exhibits good mucoadhesive properties [21]. Therefore, coating SLNs with CS may promote contact with the eye and, therefore, enhance drug adsorption, improve the stability of the nanocarriers, and aid as an effective permeation enhancer [22]. Furthermore, enhancing precorneal retention and increasing ocular availability may permit dose reduction and reduce dosing frequency [23].

The approaches used to manufacture MTZ-loaded SLNs include solvent evaporation, hot homogenization [24,25], and hot homogenization with ultrasonication [26]; however, the melt emulsification and ultrasonication (MEUS) method was used to manufacture the MTZ-loaded SLNs that were evaluated in this study. This technique is based on the principle of particle size reduction by the application of sound waves for the homogenization process and yields SLNs in the range of 80–800 nm [27]. Ultrasonic processing is rapid and highly reproducible, and the probe sonicator used is easy to clean while sample loss is negligible [28]. This method does not require the use of organic solvents, thereby avoiding the inclusion of diluents that may be toxic to ophthalmic tissues [29,30]. MEUS is considered reliable, as it produces SLNs of high quality with small droplet sizes; the use of ultrasonication can disrupt the oil and aqueous phases while facilitating the mixing of the oil droplets in water [31]. The CS-coated MTZ-loaded SLN formulations were characterized in terms of zeta potential (ZP), particle size (PS), the polydispersity index (PDI), % entrapment efficiency (% EE), and % MTZ loading (% DL). Furthermore, the osmolarity and pH of the optimized SLN formulation was adjusted to produce a formulation with a composition that would be compatible with the eye and acceptable for ocular delivery [32,33].

The objectives of this study included formulation optimization, the manufacture of CS-coated MTZ-loaded SLNs, and an investigation of the mucoadhesive properties of the product. The in vitro release of MTZ from the formulations over 10 h was investigated to facilitate a comparison with MTZ release from SLNs over 8 h [34]. The release kinetics of MTZ from the nanocarriers was evaluated by fitting experimental data to zero-order, first-order, Higuchi, Korsmeyer–Peppas, and Hixson–Crowell models using DDSolver, an add-in for Microsoft^®^ Excel [35]. The optimized SLN formulation underwent short-term stability studies and the critical quality attributes (CQA) were monitored over 28 days for products stored at 8 °C and 22 °C.

## 2. Materials and Methods

### 2.1. Materials

MTZ was purchased from Skyrun Industrial Co. Limited (Taizhou, China). Tween^®^ 20 (polysorbate 20), low-molecular-weight chitosan, acetic acid, and type II mucin from porcine stomach were purchased from Sigma Aldrich^®^ Chemical Co. (Milwaukee, WI, USA). Gelucire^®^ 48/16 (polyethylene glycol monostearate) and Transcutol^®^ HP (diethylene glycol monoethyl ether) were donated by Gattefossé SAS (Saint-Priest, France). Glycerine was purchased from Barrs Pharmaceutical Industries (Cape Town, South Africa). HPLC grade methanol (MeOH) Romil-SpSTM with a UV cut-off of 215 nm was purchased from Microsep^®^ (Gqeberha, South Africa). A RephiLe^®^ Direct-Pure UP and RO water system (Microsep^®^, Johannesburg, South Africa) was used to produce ultrapure water for use in preparing the mobile phase and for the production of MTZ-loaded optimized SLNs. All chemicals and reagents used were of analytical grade, at least, and were used without any further purification.

### 2.2. Preparation of MTZ-Loaded SLNs

SLNs were manufactured using a modified melt emulsification and ultrasonication (MEUS) method [23]. MTZ was weighed using a Model AG135 Mettler^®^ Toledo (Urdorf, Zürich, Switzerland) top-loading balance. The balance of the excipients and chemicals were weighed using a Model PA2102 OHAUS^®^ Pioneer™ precision balance (Lasec^®^, Port Elizabeth, South Africa). The lipid phase containing MTZ, Gelucire^®^ 48/16, and Transcutol^®^ HP was heated to approximately 75 °C using a Model STR-MH FMH^®^ magnetic stirrer hotplate (Labotec^®^, Johannesburg, South Africa). The aqueous phase containing Tween^®^ 20 and HPLC-grade water was heated to the same temperature, then poured into the molten lipid phase and emulsified by homogenizing the mixture at “setting one” using a Model HB989 stainless-steel hand blender (Platinum^®^, Port Elizabeth, South Africa) for one minute. The pre-emulsion was then sonicated to produce an SLN dispersion using a Sonoplus^®^ HD 4200 probe sonicator (Bandelin^®^, Berlin, Germany) fitted with a titanium flat tip (Bandelin^®^, Berlin, Germany) at a predetermined amplitude for a specific time. The SLN dispersions were then poured into amber glass bottles and allowed to cool for 24 h at room temperature (22 °C) to permit recrystallisation, after which characterization of the formulations was undertaken.

### 2.3. Preparation of CS-Coated MTZ-Loaded SLN

A 1% *w*/*v* CS stock solution was prepared by dissolving 100 mg CS in 100 mL 1% *v*/*v* acetic acid with stirring at 500 rpm at room temperature (22 °C). Thereafter, 1 mL of the 0.1% *w*/*v* chitosan solution was added dropwise to the MTZ-loaded SLN solution, with constant stirring at 300 rpm using a magnetic stirrer (Lasec^®^, Port Elizabeth, South Africa) at a temperature of 22 °C [36].

### 2.4. Characterization of CS-Coated SLN

#### 2.4.1. Particle Size (PS) and Polydispersity Index (PDI)

The PDI and PS of each CS-coated SLN formulation was determined using a Zetasizer Nano-ZS 90 (Malvern Instruments Ltd., Worcestershire, Malvern, UK) set to photon correlation spectroscopy (PCS) mode. Briefly, 30 μL of the SLN dispersion was transferred into a 10 mL volumetric flask and increased in volume with HPLC-grade water. The solution was then vortexed using a Vortex-Genie^®^ 2 vortex mixer (Bohemia, NY, USA) for 30 s before transferring into a 10 nm × 10 nm × 45 nm polystyrene cell for measurement. Ten measurements of the sample were performed at a scattering angle of 90°. The data were analyzed using Mie theory, with real and imaginary refractive indices set at 1.456 and 0.01, respectively [23].

#### 2.4.2. Zeta Potential (ZP)

The ZP of the CS-coated SLN was measured using a Zetasizer Nano-ZS 90 (Malvern Instruments Ltd., Worcestershire, UK) set to laser Doppler anemometry (LDA) mode at a wavelength of 633 nm. Prior to analysis, 30 μL of the SLN dispersion was transferred to a 10 mL volumetric flask and increased in volume with HPLC-grade water. This solution was then vortexed using a Vortex-Genie^®^ 2 vortex mixer (Bohemia, NY, USA) for 30 s. The sample was transferred into a Z-folded capillary cell and tested in replicate (*n* = 10) at an applied field strength of 20 V/cm using the Helmholtz–Smoluchowski equation (Equation (1)) [37,38].

(1) $$C=\frac{\varepsilon \zeta }{\mu \sigma_{ƒ}}$$

where

C is the streaming potential coupling coefficient,

Z is the zeta potential,

ε is the fluid permittivity,

μ is the viscosity of the fluid, and

σ_ƒ_ is the electrical conductivity.

#### 2.4.3. Transmission Electron Microscopy (TEM)

TEM was used to determine the shape of uncoated and coated nanoparticle dispersions. A drop of the CS-coated SLN aqueous dispersion was placed onto a copper grid with a carbon film and excess liquid was removed using Whatman^®^ 110 diameter filter paper (Whatman^®^ International Ltd., Maidstone, UK), after which the sample was allowed to dry at 22 °C for 24 h. The sample was visualized using a Zeiss Libra^®^ 120 TEM (Zeiss, GmbH, Oberkochen, Germany) at 22 °C with a voltage of 20 kV. The negative stain reagent used for TEM imaging was uranyl acetate.

#### 2.4.4. MTZ Loading (DL) and Encapsulation Efficiency (EE)

The % DL and EE of CS-coated MTZ-loaded SLN were evaluated using ultrafiltration with Centrisart^®^ tubes (Sartorius AG, Göttingen, Germany) fitted with a cellulose triacetate membrane of 20 kDa molecular weight cut-off. Prior to analysis, approximately 2.0 mL of the SLN formulation was transferred into the outer chamber of the Centrisart^®^ tube, after which the tubes were centrifuged for 30 min at a speed of 2500 rpm using a model HN-SII Thermo IEC bench-top centrifuge (Damon, MA, USA) to separate the aqueous phase that contained free MTZ. A solution containing free MTZ was transferred into a 10 mL A-grade volumetric flask and increased in volume with HPLC-grade methanol (MeOH) (Microsep^®^, Gqeberha, South Africa) prior to quantitation using a validated RP-HPLC method [39]. The % DL and EE were calculated using Equations 2 [40] and 3 [41].

(2) $$\mathrm{EE}=\frac{\left(\mathrm{total}\;\mathrm{amount}\;\mathrm{of}\;\mathrm{MTZ}\right)-\left(\mathrm{amount}\;\mathrm{of}\;\mathrm{free}\;\mathrm{MTZ}\right)}{\mathrm{total}\;\mathrm{amount}\;\mathrm{of}\;\mathrm{MTZ}}\times 100\%$$

(3) $$\mathrm{DL}=\frac{\left(\mathrm{total}\;\mathrm{amount}\;\mathrm{of}\;\mathrm{MTZ}\right)-\left(\mathrm{amount}\;\mathrm{of}\;\mathrm{free}\;\mathrm{MTZ}\right)}{\mathrm{total}\;\mathrm{amount}\;\mathrm{oflipid}\;\mathrm{phase}}\times 100\%$$

#### 2.4.5. Differential Scanning Calorimetry (DSC)

Prior to DSC analysis, the optimized SLNs and CS-coated SLNs were freeze-dried without a cryoprotectant, using a Vacutec™ freeze dryer (Labconco^®^ Corp, Kansas City, MO, USA) for 72 h. The freeze-dried SLNs and CS-coated SLNs were subjected to DSC analysis using a TA Model DSC 250 that was fitted with an RCS (90) refrigerated cooling system (New Castle, DE, USA). Approximately 2–5 mg of each was accurately weighed directly into TZero aluminium pans (New Castle, DE, USA) and sealed with TZero aluminium lids (New Castle, DE, USA). DSC thermograms were generated by heating the sample from 30–175 °C and, subsequently, cooling it to 30 °C, at cooling rates of 10 °C/min, while purging with nitrogen at a flow rate of 50 mL/min. The resultant data were analyzed using TRIOS software version 5.0.0.44616 (New Castle, DE, USA). DSC analyses were conducted to determine if the application of CS coating to the SLNs was successful.

#### 2.4.6. pH and Osmolarity

The pH and osmolarity of the formulations were determined, as the dispersion must have a pH of between 6.6 and 7.7 and osmolarity of between 220 and 450 mOsm/kg to ensure that the nanocarriers do not cause irritation to the eye following instillation [42,43] Glycerine (Ndabeni, Cape Town, South Africa) was used to adjust the osmolarity of the optimized CS-coated MTZ-loaded SLN formulations to a physiological value determined using freezing point depression with the aid of a calibrated Osmomat 3000 osmometer (Gonotec, Berlin, Germany). The pH of the formulations (*n* = 3) was monitored at 22 °C using a calibrated Accsen^®^ instrument pH meter (Lasec^®^, Gqeberha, South Africa).

#### 2.4.7. Mucoadhesion

Nanoparticles with a negative ZP tend to reduce ocular residence times, as any interaction between the nanocarriers and mucin, a negatively charged glycoprotein, is unlikely to happen [44]. The incorporation of CS into the SLN formulation was investigated to determine if mucoadhesive properties could be imparted [23]. The mucoadhesive properties of the CS-coated SLN were evaluated by monitoring the ZP following incubation of the CS-coated SLN with a 0.1% *w*/*w* dispersion of mucin in HPLC-grade water [45]. Mucoadhesion studies were guided by a published paper that used available material that was present in our laboratory [23]. In the investigation of the mucoadhesive properties of biopolymers, such as chitosan, ZP measurements are mostly used [46,47,48,49]. The dispersion was stirred for 30 min at 500 rpm at room temperature (25 ± 0.5 °C), using a magnetic stirrer (Monitoring and Clinical Laboratories^®^ (MCL), Johannesburg, South Africa) prior to filtration through a 0.2 μm Millipore^®^ Millex-HV Hydrophilic PVDF filter membrane (Millipore^®^ Co., Bedford, MA, USA). A 60 μL aliquot of the CS-coated SLN dispersion was injected into 20 mL of the filtered mucin dispersion and the mixture was stirred at 200 rpm for 6 h in a glass beaker maintained at 32 °C ± 0.5, using a digital hotplate stirrer (Monitoring and Clinical Laboratories^®^ (MCL), Johannesburg, South Africa) [50]. Any change in the ZP of the CS-coated carriers was likely due to the interaction of the SLNs with mucin [51]. The temperature that was used simulated the temperature of the ocular surface. The ZP of the mixture was measured at 0, 30, 60, 120, 240, and 360 min and the data were analyzed as described in Section 2.4.2. Aqueous dispersions of filtered mucin and CS-coated SLNs were also monitored and analyzed over 6 h, and these data were used as the control.

#### 2.4.8. In Vitro Release of MTZ

The in vitro release of MTZ from the CS-SLN dispersion after adjustment with glycerin was investigated, using the dialysis method [23]. A dialysis bag of 14 kDa molecular weight cut-off (Sigma Aldrich^®^ Chemical Co., Milwaukee, WI, USA) was soaked in HPLC-grade water for 12 h prior to use, after which the dialysis bag was cut open and fixed onto the opening of a glass basket with an elastic band and thread to prevent leakage of the contents from the basket [52]. Once the dialysis bag was secured in place, a 2.0 mL ± 0.5 aliquot of the optimized CS-coated SLN dispersion, after the addition of glycerine (MW = 92.09 g/mol) [53], was placed into the glass basket. HPLC-grade water was used as the desired medium for the in vitro analysis of the formulation and the pH of the HPLC water was within the pH range for ophthalmic formulations (between 6.6 and 7.8) [54]. The use of a buffer was attempted and when analysis was carried out using HPLC, crystallization in the column occurred and the experiment was reverted back to using HPLC water. The basket was then lowered into 50 mL HPLC-grade water (pH = 7.20) at a temperature of 32 ± 0.5 °C and agitated at 100 rpm, using a hotplate stirrer (Monitoring and Clinical Laboratories^®^ (MCL), Johannesburg, South Africa) at a temperature of 32 ± 0.5 °C to simulate ocular surface temperature. Approximately 0.75 mL aliquots of each sample were removed at each time interval (*n* = 3) and replaced with 0.75 mL of fresh HPLC-grade water, using a 1000 μL GILSON^®^ Pipetman (Lasec^®^, Port Elizabeth, South Africa). Samples were withdrawn at 0.5, 1.0, 2.0, 4.0, 6.0, 8.0, and 10.0 h and the 0.75 mL aliquots were analyzed using a validated RP-HPLC method specific for MTZ [39]. The in vitro release data for the CS-coated MTZ-loaded SLN were fitted to zero-order, first-order, Higuchi, Hixson–Crowell, and Korsmeyer–Peppas mathematical models to elucidate the mechanism of release and to identify the model that best fitted the release data based on the Akaike information criterion (AIC), adjusted coefficient of determination (R^2^_adj), and model selection criterion (MSC), which are the evaluation criteria for fitting dissolution data to models [35]. Identification and acceptance were based on the lowest AIC and the highest R^2^_adj and MSC.

## 3. Results

### 3.1. Preparation and Identification of a CS-Coated SLN Dispersion

A 1% *w*/*v* CS stock solution was prepared by dissolving 100 mg CS in 100 mL 1% *v*/*v* acetic acid, while stirring at 500 rpm at 22 °C. Different concentrations of 0.01% *w*/*v*, 0.03% *w*/*v*, and 0.05% *w*/*v* were prepared by pipetting 1 mL, 3 mL, and 5 mL of the stock solution, respectively, using a 1000 μL GILSON^®^ Pipetman (Lasec^®^, Port Elizabeth, South Africa) and adding dropwise to 99 mL, 97 mL, and 95 mL (100 mL batch size) of each SLN formulation with stirring at 22 °C [36]. A potential CS-coated SLN dispersion was identified following evaluation of PS, PDI, ZP, EE, DL, and pH. These data are summarized in Table 1.

Disperse systems with a PDI of 0 are considered mono-disperse, and as the PDI increases to 0.500, relatively broad particle size distributions are observed; however, they are considered acceptable [23]. A CS concentration of 0.01% *w*/*v* (Table 1) was identified for use, as the PDI was <0.50 with the pH falling within the ophthalmic range and the ZP, which was positive, may have mucoadhesion capability. Furthermore, the PS was in the nanometre dimension range and did not affect the EE of the formulation. CS concentrations of 0.03% and 0.05% were acceptable; however, they were not used, due to the low pH, and this may affect the permeation of the drug, thereby affecting the effectiveness of the formulation. An appropriate pH range for topical ophthalmic formulations should fall between 6.6 and 7.8 [54]. The pH of the formulation has a significant influence on the API permeation, and an increase in pH may result in improved permeation [55].

### 3.2. Mucoadhesion 

The interaction between CS-coated MTZ SLNs with mucin was investigated to establish whether the nanocarriers exhibited mucoadhesive properties. The inclusion of CS onto the nanoparticles has been reported to improve adhesion to the ocular surface, leading to longer retention and improved ocular bioavailability [56]. CS is considered to be a superior mucoadhesive, due to its ability to develop molecular attraction forces by electrostatic interaction with the negative surface charge of mucin [27,28].

**Figure 2:**
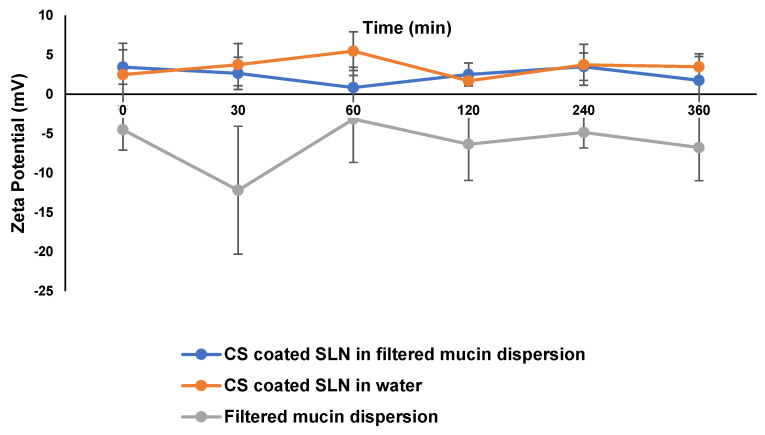
ZP of CS-coated SLN incubated in a 0.1% *m*/*v* aqueous dispersion of mucin (*n* = 10 ± SD).

Following incubation, with mucin, an increase and decrease in the magnitude of ZP were observed, as depicted in Figure 2, which implies that the CS-coated SLN formulation is likely to exhibit enhanced mucoadhesive properties when compared with the uncoated SLNs. With mucoadhesion properties imparted to the SLN dispersion, electrostatic attraction is the most likely mechanism by which the interaction occurs, as the positively charged particles are likely to interact with the negatively charged mucin.

### 3.3. Characterisation of Optimized SLN

#### 3.3.1. Particle Size

**Figure 3:**
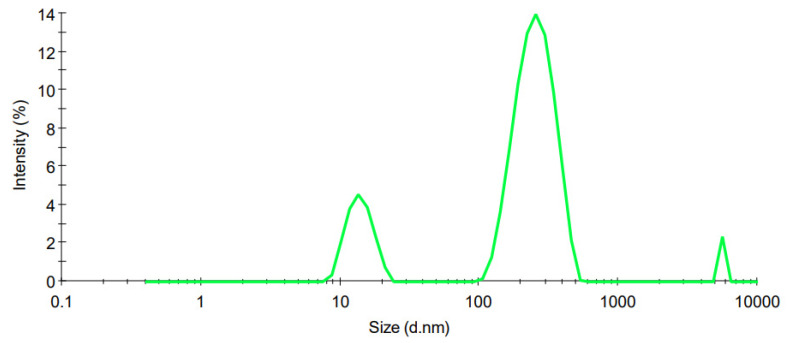
PS distribution curve for the optimized CS-coated MTZ-loaded SLN dispersion.

The PS distribution curves of the optimized CS-coated MTZ-loaded SLNs with a PS of 453.33 nm ± 74.86 and PDI of 0.437 ± 0.04, are depicted in Figure 3. The PS from the CS-coated MTZ-loaded SLN dispersion exhibits a polydisperse distribution, which may result from the process involving the separation of large particles, multiple sources of particles, or variable growth mechanisms in the formulation [57], which are normally associated with the MEUS method of production.

#### 3.3.2. Zeta Potential

**Figure 4:**
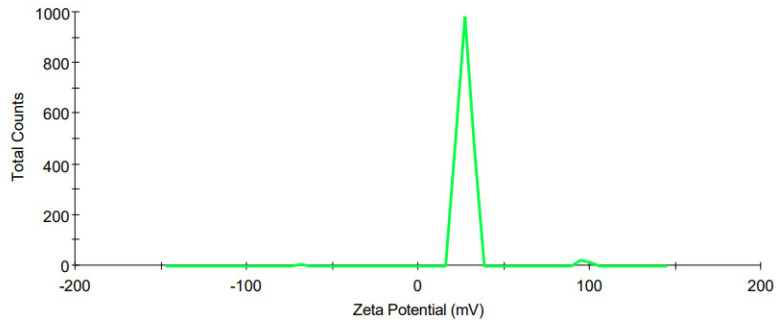
ZP distribution for the optimized CS-coated MTZ-loaded SLN.

The ZP distribution for the optimized CS-coated MTZ-loaded SLN for the ZP of +6.26 mV ± 1.09 is depicted in Figure 4. The ZP exhibits a unimodal distribution and is significant, as it determines whether the nano emulsion will exhibit short- and/or long-term stability [58]. Emulsions with a high ZP value, whether negative or positive, are electrically stabilized, as repulsive forces exceed attractive forces in such systems [58]. For low ZP values, the emulsions are likely to coagulate or flocculate and are, therefore, likely to be unstable [58]. The formulation, coagulation, or flocculation will imply that it is not suitable for administration, especially since it will be administered ophthalmically.

#### 3.3.3. Transmission Electron Microscopy (TEM)

**Figure 5:**
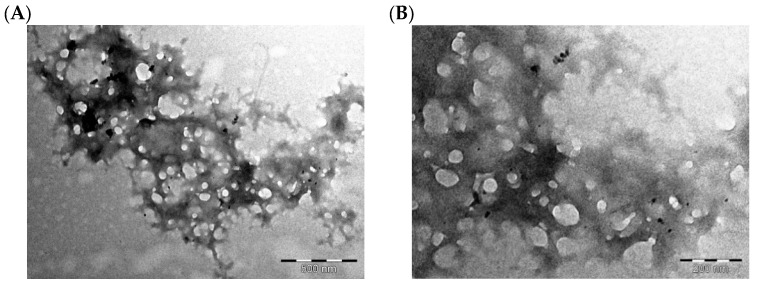
TEM micrographs of CS-coated MTZ-loaded SLNs (magnification = 500 nm (**A**) and 200 nm (**B**)).

TEM micrographs of the CS-coated MTZ-SLNs are depicted in Figure 5.

The particles were spherical in shape and in the range of nanometre size. The shape of the CS-coated MTZ-loaded SLNs may be determined by the purity of the lipid used. Chemically pure and homogenous lipids usually produce nanoparticles that appear cuboid in shape [59] and lipids that are chemically poly-disperse produce spherical particles [59,60,61]. The use of a surfactant in SLN formulations can, however, cause pure lipids to lose their crystal structure and nanoparticles morph from cuboid to spherical shapes as a result [59].

### 3.4. In Vitro Release of MTZ and Kinetic Modelling

In vitro release testing was conducted in triplicate (*n* = 3) for the optimized CS-coated MTZ-loaded SLNs over 10 h. The data were collected and fitted to mathematical models using DDSolver to determine the release kinetics. Models for which the R^2^ adjusted and MSC were the largest and for which the AIC was the lowest were identified as the best fit model for the data. The model that best described MTZ release from the particles was a first-order kinetic model (Table 2), implying that MTZ release from the SLNs was dependent on MTZ concentration and that the higher the concentration, the more rapid the release [62].

**Figure 6:**
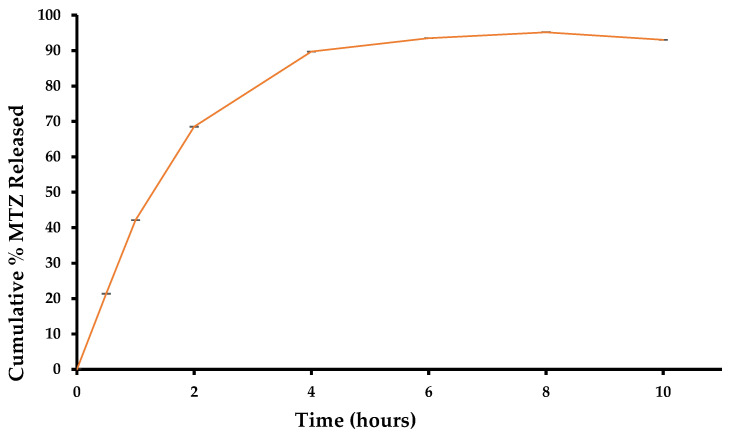
Cumulative per cent MTZ released over 10 h for optimized CS-coated SLN formulation (*n* = 3 ± SD).

The release of MTZ from the optimized CS-coated SLNs (*n* = 3) over 10 h is depicted in Figure 6, which reveals an initial burst release within the first four hours, where over 80% of the drug was released. The initial rapid release of MTZ was followed by a sustained release and suggested that a biphasic release of MTZ may have taken place, and this was observed for SLN [63]. The sustained release of MTZ from the CS-coated SLNs is a result of agitation during dissolution testing, which degrades the surfactant layer on the surface [64,65]. A cumulative per cent release of 100% was achieved over 10 h.

The initial release of MTZ should be rapid enough to ensure rapid in vivo attainment of therapeutic levels [66]. The subsequent slow release is mainly due to the slow diffusion of MTZ molecules through the lipid matrix of the nanoparticles [67,68,69]. This initial burst release that was observed suggests that a rapid delivery of MTZ to the target site is possible, with the result that bacterial growth would be inhibited, quickly improving the effectiveness of the formulation, which would need to be confirmed by undertaking microbial inhibition and/or in vivo studies.

### 3.5. CS-Coated SLN Stability Studies

#### 3.5.1. Particle Size and Polydispersity Index

**Figure 7:**
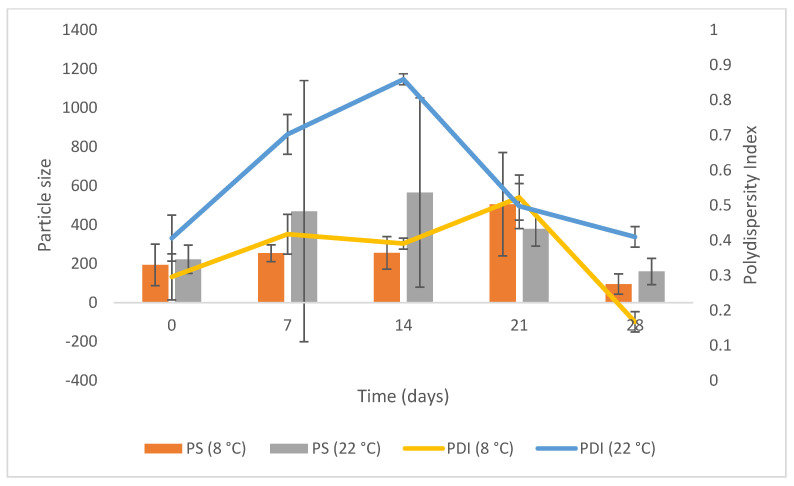
PS and PDI following storage of CS-coated MTZ-SLN for 28 days at 8 °C and 20 °C (*n* = 3 ± SD).

PS is a useful parameter that indicates the stability of a formulation, and it should remain within a narrow range when the product is stored for an extended period [69]. The PS and PDI were monitored over 28 days for products stored at 8 °C and 22 °C and the results are depicted graphically in Figure 7.

The PS at 8 °C was between 50 and 1000 nm for all samples over the test period and the PDI at 8 °C was < 0.5. The PS at 22 °C revealed a slight increase and a subsequent decline, suggesting that at higher storage temperatures, where the kinetic energy of the system increased, an increased number of collisions between particles occurred that led to agglomeration, an increase in particle size, and possible instability of the technology [70,71]. The PS increase and decrease may be attributed to a temperature-dependent factor, in which an increase in temperature causes the particles to destabilize and agglomerate, accompanied by particle destabilization [72,73]. This increase and decline affected the PDI, confirming a relationship between these two parameters. The average size of nanoparticles increases with the increase in reaction temperature [74]. In both formulations, the PS was within nanometre size and was suitable for ophthalmic use, as particles with a PS of up to 10 μm in diameter can be tolerated by the human eye [75].

#### 3.5.2. Zeta Potential

**Figure 8:**
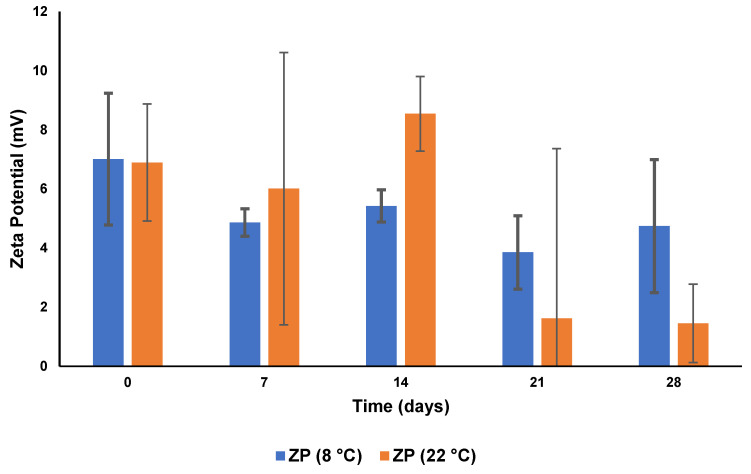
The ZP of CS-coated MTZ-SLN for 28 days following storage at 8 °C and 22 °C (*n* = 3 ± SD).

The ZP following storage for 28 days at 8 °C and at 22 °C for both formulations is depicted graphically in Figure 8.

The ZP generally decreased during the 28-day period and was more pronounced when stored at 22 °C, suggesting that the formulation was unstable and that a storage temperature of 8 °C would be required to maintain the stability of the formulation. As the formulations crystalline reorient upon storage, the particle surfaces change in charge, resulting in a change in the ZP [76].

#### 3.5.3. pH and Osmolarity

**Figure 9:**
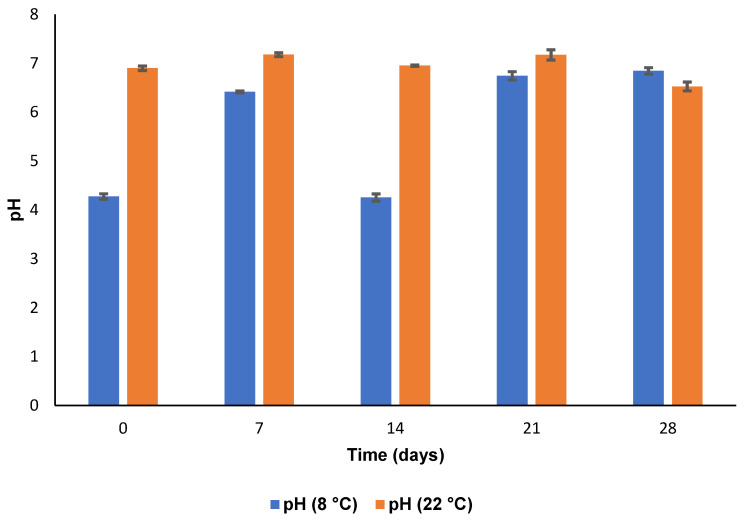
pH following storage of CS-coated MTZ-SLN for 28 days at 8 °C and 22 °C (*n* = 3 ± SD).

**Figure 10:**
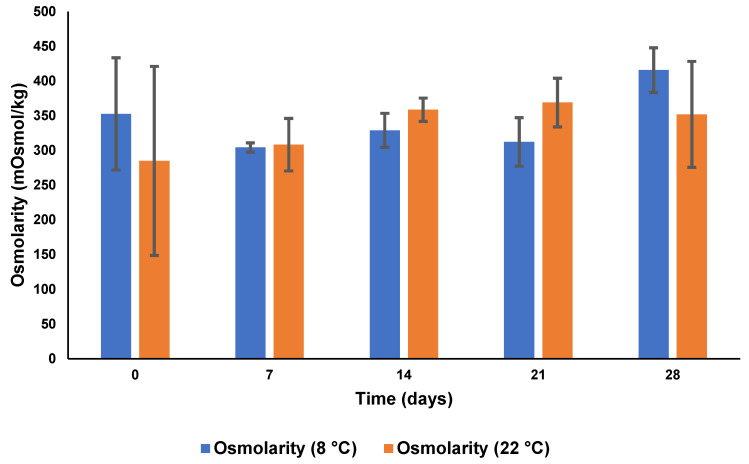
Osmolarity following storage of CS-coated MTZ-SLN for 28 days at 8 °C and 22 °C (*n* = 3 ± SD).

The pH and osmolarity data following storage at 8 °C and 22 °C for four weeks are depicted in Figure 9 and Figure 10. It is evident that the osmolarity was within an acceptable range at both 8 °C and 22 °C. The eye can tolerate a pH range of 4–8 with any pH outside of this range causing patient discomfort and irritation to ocular tissues which, in turn, may result in a decrease in bioavailability because of increased lachrymal secretions [77]. The pH was within the normal range throughout the test period. Therefore, the CS-coated MTZ-loaded SLN formulation was considered suitable for ophthalmic delivery in the context of pH and osmolarity.

## 4. Conclusions

CS-coated MTZ-loaded SLN could be a potential formulation for ophthalmic delivery and MTZ release was achieved over 10 h have been produced. The mucoadhesive properties of the optimized CS-coated MTZ-loaded SLN may increase ocular availability, enhance precorneal retention, and permit dose reduction whilst using longer dosing frequencies.

MEUS was used as the manufacturing approach as it is a relatively simple and low-cost method [78] and the inconsistent availability of municipal water is vital when using hot high-pressure homogenization (HHPH) [79]. However, this technique would be too costly to scale up for high production of CS-coated MTZ-loaded SLN.

Although stability studies revealed a decrease in the PS and PDI over the 28-day test period at 8 °C and 22 °C, the PS remained within the nanometre size range. The decrease in ZP was mitigated to some extent since the presence of polysorbate provides stability through both electrostatic and steric hindrance mechanisms [76]. The pH and osmolarity were in the range 4–8 and 220–450 mOsm/kg, respectively, and are at levels which are well tolerated by the human eye.

Formulating a powder for reconstitution as a single dose may further enhance the stability of the formulation and the incorporation of an in-situ gel may increase the viscosity and contact time of the formulation with the eye, dealing with potential adherence issues. With further optimization of the formulation, in the future, cytotoxicity and in vivo studies must be conducted on the CS-coated MTZ-loaded SLN to ensure safety in animal models prior to initiating randomized controlled trials in humans. As the MEUS technique may not be a good option for the high production of CS-coated MTZ-SLN, future consideration would include the exploration of a sustainable production approach.

## Figures and Tables

**Table 1:** Summary of results for potential CS-coated SLN dispersions.

CS Concentration in SLN Dispersion (% *w*/*v*)	PS (nm)	ZP (mV)	PDI	EE (%)	DL (%)	pH
**0.01**	**453.33 ± 74.86**	**6.67 ± 0.40**	**0.43 ± 0.04**	**99.91**	**4.99**	**6.72 ± 0.03**
0.03	274.27 ± 141.31	7.39 ± 2.98	0.37 ± 0.10	99.91	4.99	4.81 ± 0.02
0.05	247.69 ± 223.69	4.9867 ± 2.25	0.37 ± 0.16	99.90	4.99	4.40 ± 0.01

**Table 2:** Summary of model kinetic data for the release of CS-coated MTZ-loaded SLNs.

Model and Equation	Code Evaluation Criteria		
	R^2^_adj_	AIC	MSC
Zero-order (A) $\left(F=k0\times t\right)$	0.3892	71.5384	−0.2779
**First order (B)** (F=100×[1−Exp(−k1×t)])	**0.9924**	**36.449**	**4.1083**
Higuchi (C) F=kH×t^0.5	0.8789	58.5922	1.3404
Korsmeyer–Peppas (D) F=kKP×t^n	0.9164	56.3928	1.6153
Hixson–Crowell (E) F=100×1−1−kHC×t ^3	0.9701	47.3949	2.74

## Data Availability

Not applicable.
